# Artificial Intelligence (AI) Supported Decision-Making in Intensive Care Units: Implications for Nursing and Medical Practice

**DOI:** 10.7759/cureus.104266

**Published:** 2026-02-25

**Authors:** Sumangal Bose, Avinash Prakash, Avijit Kumar Prusty, Rashmi Verma, Karthika Padmavathy, Venugopal Reddy Iragamreddy

**Affiliations:** 1 Hospital Administration, Sanjay Gandhi Postgraduate Institute of Medical Sciences, Lucknow, IND; 2 Anesthesiology, All India Institute of Medical Sciences (AIIMS) Nagpur, Nagpur, IND; 3 Intensive Care Unit, Critical Care Shanti Memorial Hospital, Cuttack, IND; 4 Trauma and Emergency, All India Institute of Medical Sciences (AIIMS) Bhopal, Bhopal, IND; 5 Pathology, Sri Lalithambigai Medical College and Hospital, Dr M.G.R Educational and Research Institute, Chennai, IND; 6 Paediatrics, Ovum Woman and Child Speciality Hospital, Bangalore, IND

**Keywords:** artificial intelligence, clinical decision-making, critical care, intensive care units, nursing practice

## Abstract

Artificial intelligence (AI) is transforming intensive care medicine by enabling data-driven, real-time decision-making in complex and high-acuity clinical environments. However, its rapid incorporation into healthcare presents profound ethical, clinical, and professional challenges that warrant comprehensive evaluation. This structured narrative review synthesises literature published between 2015 and 2025 to explore how artificial intelligence supports diagnostic, prognostic, monitoring, and therapeutic decision-making in intensive care units (ICUs) and to assess its implications for nursing and medical practice. The findings reveal that AI enhances diagnostic precision, predictive accuracy, and workflow efficiency while improving patient safety and optimising resource utilisation. Nonetheless, ongoing concerns about interpretability, accountability, data quality, and algorithmic bias underscore the necessity for transparent governance, ethical oversight, and multidisciplinary collaboration. Distinctively, this review integrates technological, ethical, and interprofessional perspectives to present a holistic framework for understanding human-artificial intelligence collaboration in critical care. It emphasises that sustainable adoption depends on explainable, context-sensitive systems, clinician engagement, and the inclusion of AI literacy in professional training. This review advances the discourse by framing AI not as a replacement but as a transformative partner, arguing that the future of intensive care lies in harmonising computational precision with clinical empathy to deliver ethical, equitable, and patient-centred outcomes.

## Introduction and background

The intensive care unit (ICU) is one of the most technologically advanced and data-intensive environments in modern healthcare. Clinicians and nurses must make high-stakes decisions rapidly while continuously monitoring patients’ physiological status [1]. Critical illness is dynamic and unpredictable, requiring real-time interpretation of large volumes of data, including vital signs, laboratory results, imaging findings, and patient history [2,3]. These conditions contribute to cognitive overload and decision fatigue, increasing the risk of error [4]. Within this context, artificial intelligence (AI) has emerged as a data-driven decision-support approach aimed at enhancing clinical judgment and patient outcomes in the ICU [5].

AI refers to computational systems capable of performing tasks that typically require human cognition, including pattern recognition and predictive reasoning [6]. Early medical AI applications in the 1970s and 1980s relied on rule-based expert systems such as MYCIN, but their clinical impact was limited by restricted computational capacity and data availability [7]. Advances in electronic health records (EHRs), big data analytics, and machine learning have enabled practical implementation of AI in contemporary ICU settings [8]. Current applications include early sepsis detection, ventilator management, prediction of patient deterioration, and precision dosing support [9].

**Figure 1:**
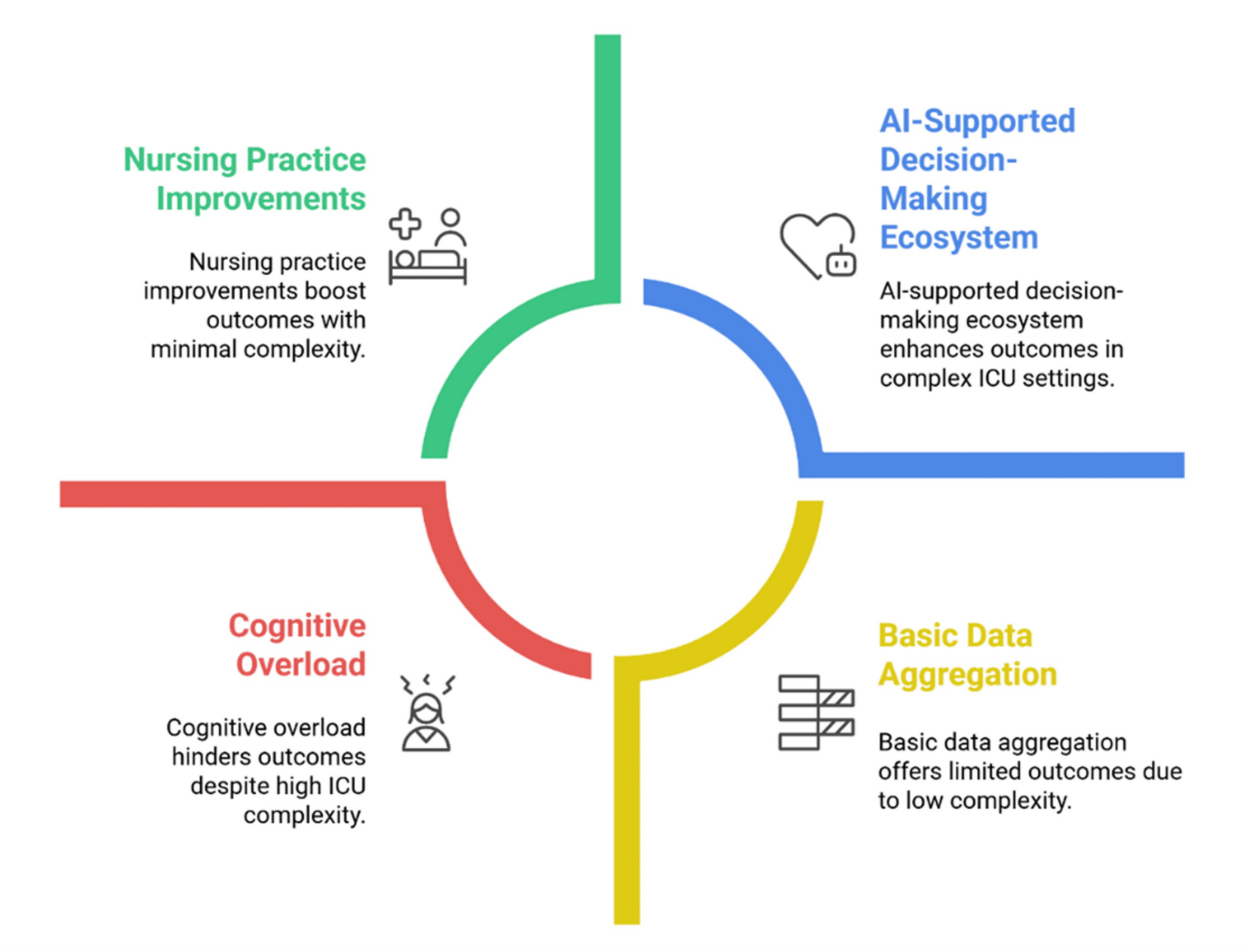
Key Components Influencing AI-Supported Decision-Making in Intensive Care Units Created by authors based on [1,4,10,15]

The ICU generates high-dimensional and heterogeneous data streams [10], with individual patients producing thousands of physiological data points daily [11]. Machine learning models can identify nonlinear patterns and latent physiological signals that may not be readily apparent to clinicians [12,13]. Beyond individual patient monitoring, AI also contributes to operational efficiency through workflow optimisation and resource allocation [14]. Thus, AI functions not merely as a technological adjunct but as a system-level approach to improving safety, quality, and continuity of care [15]. Figure 1 illustrates how AI-supported decision-making integrates data analytics with clinical collaboration to enhance patient outcomes.

However, AI implementation in ICU decision-making raises important clinical and ethical considerations [16]. Clinical decisions are highly context-specific and often informed by experiential judgment (gestalt) shaped by pattern recognition and situational awareness [17]. AI may enhance consistency and predictive accuracy, but it cannot replace value-based human reasoning [18]. Limited interpretability of some AI models (“black-box” systems) restricts clinicians’ ability to understand how outputs are generated, potentially reducing trust and adoption [19]. Ethical deployment in life-critical settings, therefore, requires transparency, accountability, and clearly defined human oversight [20]. Effective human-AI collaboration must prioritise augmentation of clinical expertise rather than substitution [11]. 

In the case of nursing professionals, AI presents both opportunities and challenges [20]. Nurses play a central role in continuous patient monitoring, documentation, and early recognition of clinical deterioration [7]. By automating parts of data interpretation and surveillance, AI can reduce routine cognitive burden and allow nurses to focus more on direct patient care, communication, and emotional support [8]. However, this transformation requires new competencies, including digital literacy, basic data interpretation, and human-machine interaction skills [17]. It also raises ethical concerns related to professional autonomy, responsibility, and the preservation of compassionate care within technology-mediated environments [21]. Accordingly, nursing education and institutional policy must integrate AI-related competencies while maintaining humanistic principles of care [1].

From a medical perspective, AI can strengthen clinical reasoning, improve diagnostic accuracy, and support personalised therapeutic decisions [15]. Natural language processing tools can extract clinically relevant information from unstructured notes, while decision-support systems can synthesise real-time patient data to generate evidence-informed recommendations [13]. Empirical evidence suggests that such systems may reduce diagnostic variation, streamline workflows, and improve interprofessional coordination [22]. However, successful implementation depends on high-quality data, interoperability across digital systems, and active clinician involvement in model development, validation, and governance [19].

Evolution of AI in critical care

The evolution of artificial intelligence in critical care reflects the broader transformation of healthcare toward data-driven and computationally supported practice [23]. The intensive care unit, characterised by continuous monitoring, rapid intervention, and complex decision-making, has become a primary environment for AI implementation [24,25].

During the early twenty-first century, advances in machine learning and deep learning marked a transition from static decision-support tools to dynamic, data-driven predictive models capable of analysing high-dimensional ICU datasets [26,27]. These approaches enabled systems to learn directly from large-scale clinical data rather than relying solely on predefined logic. Progress in neural networks, reinforcement learning, and unsupervised modelling has facilitated the detection of subtle physiological signatures associated with patient deterioration [28]. For example, deep learning architectures have demonstrated the capacity to identify early indicators of sepsis, acute respiratory distress syndrome, and cardiac arrest before overt clinical manifestation, allowing earlier intervention [29].

More recently, AI development has shifted toward integrated clinical ecosystems rather than isolated predictive algorithms [24]. In high-income healthcare settings, AI tools are increasingly embedded within electronic health record platforms through dashboards, automated alerts, and centralised monitoring infrastructures, whereas resource-constrained environments often rely on cloud-based or open-source implementations [30]. Despite ongoing technological advancement, challenges related to interpretability, equity, accountability, and clinician trust remain central to responsible implementation [23]. AI continues to evolve as a decision-support adjunct designed to augment, rather than replace, human clinical judgment [22].

Objectives of the review

This review seeks to examine how AI has been used in a complex position to aid decision-making in intensive care units, with a focus on its impact on nursing as well as medical practice. It addresses the continuum of AI applications between predictive analytics and clinical decision support systems and determines their effects on clinical performance, safety, and the dynamics of the workflow. In addition, it identifies ethical, legal, and organisational issues that come with the implementation of AI and argues for the necessity of clear, explainable, and context-specific algorithms. Finally, the paper will suggest that the future of AI in critical care is not to oust human decision-makers but to establish a mutually beneficial relationship where human judgment and machine intelligence are combined to improve patient care.

Methodology

Study Design

This study employed a structured narrative review design to examine the application of AI in decision-making within ICUs and its implications for nursing and medical practice. The review focused on literature published between January 2015 and December 2025 to capture contemporary developments in AI implementation in critical care.

Search Strategy

A structured literature search was conducted in PubMed, Scopus, Web of Science, CINAHL, IEEE Xplore, and ScienceDirect to ensure comprehensive coverage of medical, nursing, and computational research. The search strategy combined controlled vocabulary terms (e.g., MeSH in PubMed) and free-text keywords using Boolean operators (AND, OR). The primary search structure was: (“artificial intelligence” OR “machine learning” OR “deep learning” OR “neural network*” OR “predictive analytic*”) AND (“intensive care unit” OR ICU OR “critical care”) AND (“decision support” OR “clinical decision-making” OR “medical decision-making” OR “nursing practice” OR “early warning system*”). Search syntax was adapted according to the indexing requirements of each database. Reference lists of selected studies were also manually screened to identify additional relevant publications.

Eligibility Criteria and Study Selection

Eligible studies were peer-reviewed articles published in English between 2015 and 2025 that examined AI, machine learning, or deep learning applications within ICU or critical-care settings. Studies were included if they addressed diagnostic, prognostic, monitoring, therapeutic, workflow, or decision-support functions relevant to nursing or medical practice. Studies were excluded if they did not align with the aims of the review, were not conducted in ICU or critical-care settings, did not provide clinically relevant applications, or lacked sufficient methodological detail to support meaningful interpretation.

Screening was conducted in two stages, beginning with title and abstract review, followed by full-text assessment of potentially eligible studies. Selection decisions were guided by predefined inclusion and exclusion criteria to maintain consistency. As this study followed a narrative review framework rather than a systematic review protocol, a Preferred Reporting Items for Systematic Reviews and Meta-Analyses (PRISMA) flow diagram and formal risk-of-bias assessment were not undertaken.

Data Extraction and Synthesis

Data from included studies were extracted into a structured summary format capturing study design, AI methodology, clinical application, reported outcomes, and implementation considerations. A narrative thematic synthesis approach was used to integrate findings across heterogeneous study designs and disciplinary perspectives. Studies were organised into thematic domains, including AI-driven clinical decision support systems, predictive monitoring and early warning systems, system-level integration and workflow transformation, ethical and governance considerations, and implications for nursing and medical practice. Findings were synthesised descriptively to identify recurring patterns, reported benefits, implementation barriers, and professional implications. Although a formal risk-of-bias tool was not applied due to methodological heterogeneity, emphasis was placed on peer-reviewed empirical studies and clinically validated applications where available.

## Review

AI-driven Clinical Decision Support Systems (CDSS)

One of the most clinically significant applications of AI in intensive care is the use of Clinical Decision Support Systems (CDSS) [27]. These systems integrate real-time patient data with algorithmic models to support clinicians and nurses in diagnosis, prognosis, and therapeutic planning [10]. Unlike earlier rule-based systems, contemporary AI-enabled CDSS utilise machine learning, natural language processing (NLP), and real-time analytics to generate adaptive recommendations that respond dynamically to changes in a patient’s clinical status [31].

Empirical evidence highlights the contribution of AI-based CDSS to early detection and timely intervention [28]. Predictive models such as InSight and the Epic Sepsis Model generate automated risk scores within electronic health record (EHR) systems, enabling earlier identification of sepsis compared with conventional clinical recognition [32]. In practice, these tools may appear as interruptive pop-up alerts requiring acknowledgment or as continuously updated dashboard indicators, with variability in visibility and workflow integration across institutions [25]. Reinforcement learning approaches have been applied to optimise ventilator management by balancing oxygenation effectiveness with lung-protective strategies. At the same time, AI-assisted dosing systems improve precision in titrating vasoactive medications and sedatives [16].

Despite demonstrated benefits, adoption challenges persist [33]. Limited interpretability of complex deep learning models, commonly referred to as the “black-box” problem, reduces clinician confidence in automated recommendations [30,31]. Integration of CDSS outputs into existing EHR infrastructures and clinical workflows remains technically and operationally demanding [34]. Ethical concerns regarding accountability remain unresolved, particularly when AI-generated recommendations contribute to adverse outcomes and responsibility is unclear [19]. For example, if a sepsis alert fails to trigger or triggers too late and a patient deteriorates, harm may be attributed to model error, incomplete EHR data, delayed clinical response, or inappropriate system deployment [35]. Nevertheless, explainable and context-aware CDSS demonstrate potential to reduce diagnostic variability, improve timeliness of intervention, and enhance patient safety in critical care environments [36]. Table 1 summarises evidence indicating that AI-based CDSS enhance clinical accuracy and safety through predictive, adaptive, and workflow-integrated decision support mechanisms.

**Table 1:** Evolution and Clinical Impact of AI-Based Clinical Decision Support Systems in Intensive Care CDSS: Clinical Decision Support Systems.

CDSS Type / Model	Algorithmic Approach	Primary Clinical Application	Observed Benefits / Key Outcomes	Challenges / Limitations	Reference(s)
Early CDSS (e.g., rule-based systems)	Manual encoding of clinical guidelines and fixed rule sets	Diagnostic and therapeutic assistance	Provided basic standardisation of care; limited adaptability	Static reasoning; lack of real-time data integration	[21]
InSight Model	Predictive Machine Learning	Early sepsis detection	Identifies physiological patterns up to 6 hours before clinical manifestation; improves survival rates	Dependent on data quality and integration	[28]
Epic Sepsis Model	Deep Learning (pattern recognition)	Sepsis prediction and early warning	Enhances timely diagnosis and reduces mortality	Limited model transparency; variable performance across institutions	[5]
Reinforcement Learning Systems	Adaptive optimisation algorithms	Ventilator management	Balances oxygenation and lung protection through dynamic adjustments	Requires large training datasets; interpretability issues	[16]
AI-Assisted Dosing Algorithms	Machine Learning and predictive control	Precision titration of vasoactive drugs and sedatives	Improves dosing accuracy and safety; supports personalised therapy	Integration into workflows remains limited	[27]
Explainable and Context-Aware CDSS	Hybrid ML with interpretable frameworks	Comprehensive decision support (diagnosis, prognosis, therapy)	Reduces diagnostic variability; enhances clinician trust and safety	Ongoing ethical and accountability concerns	[29]

Predictive analytics and system-level integration

AI implementation in critical care has expanded rapidly, although adoption remains uneven across healthcare systems globally [37]. High-income settings increasingly integrate AI tools into EHR platforms through dashboards, automated risk scores, and real-time clinical alerts, whereas resource-constrained environments often rely on cloud-based, open-source, or standalone solutions [38]. Current development trends indicate a shift from isolated predictive algorithms toward integrated AI ecosystems that support clinicians, nurses, and administrators across clinical, operational, and safety domains [39]. Despite technological progress, interpretability, equity, accountability, and clinician trust remain significant barriers to safe and sustainable implementation in ICU decision-making [40]. AI therefore functions most effectively as a clinical adjunct that strengthens, rather than replaces, expert human judgment [41].

AI in monitoring and early warning systems

Continuous monitoring is central to intensive care, where rapid physiological changes may signal impending deterioration [42]. Conventional monitoring systems rely on fixed threshold alerts based on vital signs such as heart rate, blood pressure, and oxygen saturation [43]. Although effective, these rule-based systems frequently generate high false-positive rates, contributing to alarm fatigue and response desensitisation among clinicians [44]. AI-enhanced monitoring introduces predictive surveillance by identifying patterns associated with clinical instability before overt deterioration occurs [45].

AI-based surveillance systems integrate multimodal data, including electrocardiograms, hemodynamic waveforms, laboratory results, and imaging findings, to detect complex relationships that may not be evident when variables are assessed independently [21]. Machine learning models can predict sepsis or respiratory failure hours before traditional scoring systems identify abnormalities, based on nonlinear trends across multiple physiological parameters [46]. Neural network-based anomaly detection systems continuously analyse electrocardiographic and oxygenation signals to anticipate arrhythmias or hypoxemic events, enabling earlier intervention [28].

Advances in smart-ICU infrastructures extend these capabilities to system-level coordination [38]. Centralised command centres equipped with AI dashboards can monitor multiple patients simultaneously, prioritising those at highest risk and supporting triage and staffing decisions [47]. Wearable sensors and Internet-of-Things (IoT) devices further enhance surveillance by transmitting continuous data streams to predictive algorithms that dynamically update risk estimates [48]. These developments have demonstrated potential to reduce adverse events, improve workflow efficiency, and optimise resource allocation, particularly during periods of high demand [26].

**Figure 2:**
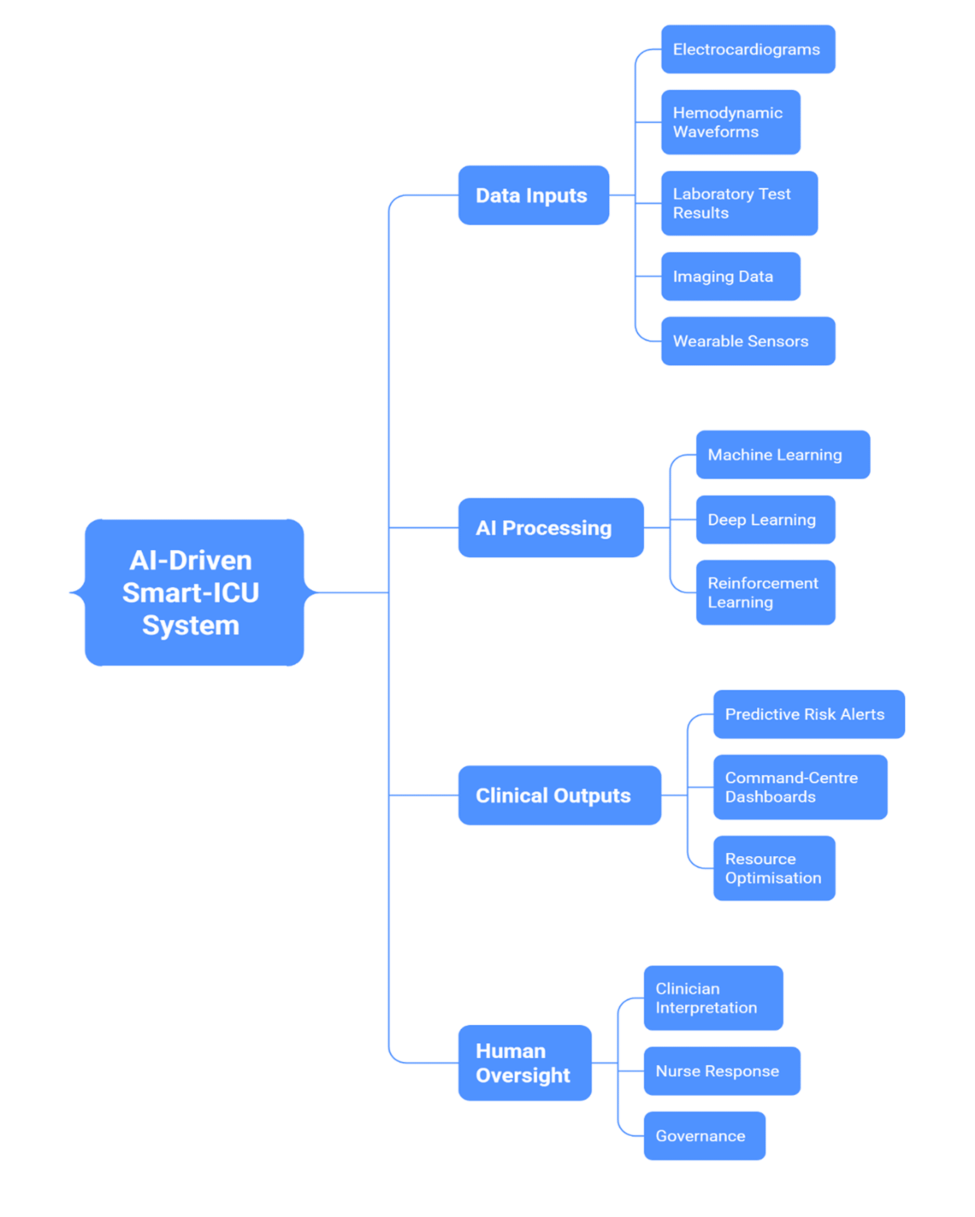
AI-Driven Predictive Monitoring Framework in Intensive Care Units Created by authors based on [16,21,28,38,46,47,49]

Implementation challenges remain [18]. Model reliability may be affected by data heterogeneity, signal noise, and interoperability limitations across digital systems [34]. Clinicians require explainable outputs to ensure that algorithmic alerts align with physiological reasoning [49]. Transparent interface design and workflow integration are essential to support user acceptance and sustained adoption [16]. When appropriately implemented, AI shifts ICU monitoring from reactive threshold-based alerting toward anticipatory risk management while maintaining human clinical accountability [40]. Figure 2 illustrates this transition from reactive alerting to predictive, coordinated surveillance.

Implications for nursing practice

Nurses are continuously present in the ICU and translate physiological information into bedside action [41]. AI-supported workflows increasingly assist by automating data synthesis and prioritisation, enabling nurses to focus more on direct patient care, communication, and clinical judgment [18]. Predictive dashboards and decision-support interfaces consolidate multiple inputs into actionable indicators, supporting earlier identification of at-risk patients and more timely escalation [27].

AI-assisted tools may reduce documentation burden and cognitive load by automatically tracking physiological trends and generating clinical summaries [12]. This can strengthen situational awareness and support timely intervention [42]. AI does not replace nursing expertise; rather, it complements complex clinical reasoning by providing structured, data-driven support [28]. For example, algorithmic guidance on fluid balance or sedation trends may support nurses in evaluating interventions and communicating risk more effectively within the care team [19].

However, AI-supported practice requires new competencies [25]. Nurses must develop digital literacy, including the ability to interpret algorithm outputs, recognise data abnormalities, and understand system limitations [20]. Investments in AI-related curricula, clinical governance, and ethical decision-making should therefore be incorporated into training and continuing professional development [43]. Accountability concerns also require attention, as AI involvement in clinical decisions may complicate responsibility attribution [17]. Maintaining trust requires clear reinforcement that final clinical judgment remains with human professionals [31].

AI adoption also influences interprofessional relationships [24]. Early warning systems may enable nurses to initiate escalation earlier, strengthening their role in patient advocacy and proactive intervention [32]. This reinforces the need for collaborative communication structures that integrate nursing expertise with interpretation of AI-supported recommendations [29]. Sustainable implementation depends on parallel development of training, governance, and ethical safeguards [44].

Implications for medical practice

For physicians, integration of AI influences diagnostic reasoning, prognostic assessment, and therapeutic precision [45]. Intensive care medicine requires continuous synthesis of large, dynamic datasets, and AI-based real-time analytics can support this process by generating evidence-informed suggestions and risk stratifications [19]. Natural language processing tools can extract clinically relevant information from unstructured notes and imaging reports, while predictive algorithms assist in triage decisions, therapy adjustments, and anticipation of complications [28].

AI systems have demonstrated high accuracy in interpreting radiological and physiological data, in some contexts approaching expert-level performance [37]. Deep learning models can detect pulmonary infiltrates, ventilator-associated complications, and subtle electrocardiographic abnormalities with high sensitivity [46]. Reinforcement learning approaches further support optimisation of vasopressor and fluid management by adapting recommendations to individual patient trajectories [27]. These applications may enhance diagnostic consistency and reduce unwarranted variability in clinical decision-making [31].

AI also influences workflow organisation [24]. Predictive dashboards support resource allocation, reduce unnecessary investigations, and may contribute to shorter ICU stays [47]. Shared AI-enabled platforms facilitate interdisciplinary communication through integrated data visualisation accessible to physicians, nurses, and pharmacists [29]. However, final clinical decisions remain the ethical responsibility of physicians, who must critically evaluate algorithmic outputs before implementation [16,44].

The physician-patient relationship is likewise affected by AI-supported decision-making [25]. Transparency regarding the role of AI in clinical judgments is essential to maintain patient trust and informed consent [35]. Attention must also be directed toward identifying and mitigating algorithmic bias to prevent reinforcement of demographic disparities [39]. Sustainable integration of AI in intensive care requires a complementary model in which machine-supported precision enhances, rather than substitutes, human clinical judgment, ethical reasoning, and contextual interpretation [48,50]. Table 2 summarises the impact of AI integration on diagnostic precision, workflow efficiency, and ethical governance in medical practice.

**Table 2:** Clinical and Operational Impact of AI Integration in Physician Practice within Intensive Care Units NLP: natural language processing, CNN: convolutional neural networks, ECG: electrocardiogram.

AI Application	Algorithmic Approach	Primary Physician Function Supported	Key Outcomes	Challenges	References
NLP-Based Information Extraction Tools	NLP (text and image mining)	Extracting insights from clinical notes and imaging reports	Enables rapid data synthesis, supports diagnosis and triage decisions	Requires high-quality data and domain adaptation	[19]
Deep-Learning Diagnostic Models	CNNs and image analysis	Detection of pulmonary infiltrates, ECG anomalies, and ventilator-associated complications	Achieves sensitivity comparable to expert clinicians; improves diagnostic precision	Limited interpretability (“black-box”)	[37]
Reinforcement Learning Systems	Adaptive optimisation and feedback algorithms	Optimisation of vasopressor and fluid dosing	Personalised therapy adjustment enhances therapeutic precision and safety.	Demands continuous data and validation across patient types	[27]
Predictive Dashboards and Workflow Optimisation Tools	Predictive analytics and real-time modelling	Resource allocation and ICU workflow coordination	Reduces unnecessary testing, shortens ICU stay, and improves resource use	Implementation cost and interoperability issues	[4]
Interdisciplinary Shared Platforms	Integrated AI-driven data visualisation	Facilitates communication among physicians, nurses, and pharmacists	Promotes collaborative decision-making and a unified clinical view	Data governance and accountability remain concerns	[16]
AI-Augmented Physician–Patient Interaction	Explainable AI and bias-detection algorithms	Enhancing transparency and patient trust in AI-mediated care	Strengthens communication, supports ethical care, and mitigates bias	Risk of algorithmic inequities and overreliance	[25]
Symbiotic AI–Physician Collaboration Models	Hybrid human–machine decision frameworks	Integrated clinical reasoning and ethical oversight	Combines machine precision with human empathy and critical judgment	Requires ethical literacy and sustained human validation	[48]

Ethical and legal considerations

Implementation of AI in ICUs introduces ethical challenges related to accountability, transparency, and justice in life-critical decision-making [14]. Although AI can improve diagnostic timeliness and accuracy through analysis of large clinical datasets, it also complicates responsibility attribution and ethical oversight [27]. Ethical integration, therefore, requires alignment of technological capabilities with established clinical principles, including beneficence, autonomy, and justice [19]. Data governance and patient autonomy represent central concerns [30]. AI models rely on large-scale data from electronic health records, bedside monitors, and wearable sensors [22]. Although datasets are often anonymised, re-identification risks persist, particularly in multi-institutional or cross-national data sharing contexts [15]. Patients may also be unaware that their health information can be used for algorithm development beyond direct clinical care, creating tension between population-level benefit and individual consent [25]. Clear governance frameworks and transparent consent mechanisms are therefore necessary to protect autonomy while enabling responsible innovation [12].

Distributive justice and algorithmic bias constitute additional barriers [29]. Models trained primarily in high-resource environments may perform poorly in low-resource ICUs, potentially reinforcing inequities in clinical outcomes [38]. Addressing bias requires diverse training datasets, population-specific validation, and regulatory oversight that evaluates fairness in addition to technical performance [18]. Explainability and accountability remain critical for safe clinical adoption [21]. Deep learning systems are frequently difficult to interpret, limiting clinicians’ ability to understand the rationale underlying model outputs [33]. In critical care settings, clinicians must be able to evaluate whether algorithmic recommendations are physiologically plausible to remain ethically and professionally accountable [36]. Emerging regulatory efforts, including the European Union AI Act and United States Food and Drug Administration (FDA) guidance on adaptive algorithms, represent early attempts to clarify standards for transparency, safety monitoring, and post-deployment responsibility [32]. Ethical deployment, therefore, depends on maintaining human agency through governance mechanisms such as ethics-by-design, ongoing model surveillance, and routine algorithm audits to ensure alignment with fairness, compassion, and professional accountability [17,26,40].

AI and interprofessional collaboration in the ICU

Adoption of AI alters the social and organisational dynamics of intensive care by reshaping how clinicians and nurses interact with information and with one another [12]. Critical care has always relied on collaborative practice, and AI now functions as an additional analytic layer that consolidates data, generates forecasts, and supports collective interpretation [28]. Shared dashboards and predictive interfaces provide synchronised, real-time access to patient information, strengthening situational awareness and evidence-based discussion during multidisciplinary rounds [19,37,45]. For example, early-warning alerts for sepsis may be simultaneously visible to nurses and physicians, promoting coordinated and timely response [30].

AI integration also modifies communication patterns within the team [23]. Nurses may receive and interpret algorithm-generated alerts before physicians review them, potentially accelerating intervention but also altering traditional hierarchies of authority [10,32]. Clear response protocols and interprofessional training are therefore necessary to define roles and responsibilities in AI-supported workflows [41]. Effective implementation depends on mutual trust: physicians must recognise nursing contributions to data interpretation, and nurses must feel empowered to act while maintaining collaborative oversight [14].

**Figure 3:**
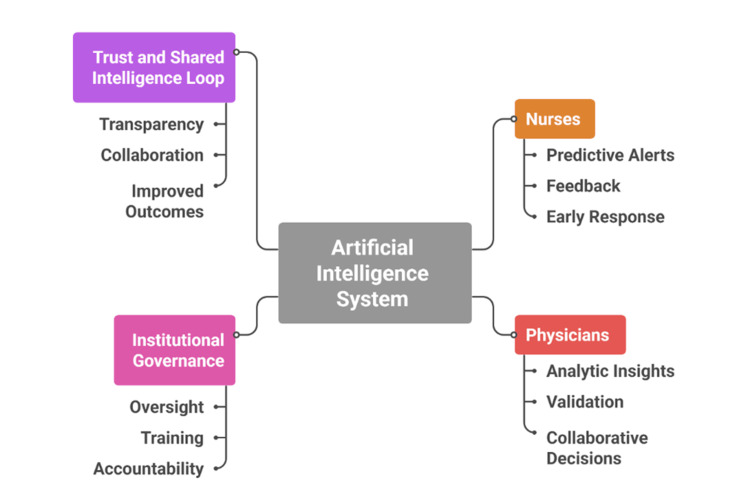
Interprofessional Collaboration Framework in AI-Enabled Intensive Care Units Created by authors based on [16,19,26,42]

Trust extends beyond interpersonal dynamics to confidence in the AI systems themselves [33]. Sustained reliability, transparent outputs, and feedback mechanisms that allow clinicians to question or refine algorithmic recommendations are essential for adoption [22]. When appropriately governed, AI can redistribute cognitive workload across the team, supporting a model of shared intelligence that combines human clinical judgment with computational analysis [46]. Development of this model requires interprofessional culture-building, joint simulation training, participatory model evaluation, and inclusive governance structures to ensure that technology strengthens rather than disrupts collaborative care [29,49]. Figure 3 illustrates how AI enhances information transparency, reshapes communication pathways, and supports trust within ICU teams.

Challenges in implementation

Although the conceptual benefits of AI in ICUs are increasingly recognised, real-world implementation remains constrained by technical, organisational, economic, and regulatory barriers [29]. Data integrity is a foundational limitation, as ICU datasets are often heterogeneous, asynchronous, and incomplete [46]. Device calibration inconsistencies, documentation errors, and limited interoperability across monitoring systems can degrade model performance and reduce reliability [24]. Many hospital information systems also remain siloed, restricting the continuous data flow required for robust machine learning deployment [33]. Standardised data formats and interoperable digital infrastructures are therefore prerequisites for dependable clinical implementation [42].

Human and organisational factors further influence adoption [20]. Clinicians may resist AI integration due to concerns about autonomy, uncertainty regarding system reliability, or lack of familiarity with AI-supported workflows [41]. Conversely, excessive reliance may lead to automation bias, where users accept algorithmic outputs despite conflict with clinical judgment [26]. Sustainable implementation, therefore, requires calibrated trust supported by critical appraisal, transparent communication, and structured change-management strategies [35,44].

Economic and logistical barriers also limit scalability [23]. Deployment often requires not only software acquisition but also hardware upgrades, cybersecurity capacity, and ongoing model retraining using local datasets [48]. Resource-constrained institutions may lack these capabilities, increasing the risk of a digital divide in critical care quality [31]. Evaluation practices represent an additional challenge, as many studies prioritise performance metrics such as accuracy or area under the curve without adequately assessing usability, workflow compatibility, and patient-centred outcomes [38,45]. Lack of standardised evaluation frameworks limits inter-institutional comparison and complicates evidence-based policymaking [25]. International collaboration and benchmarking initiatives are therefore needed to assess both technical performance and real-world clinical impact [49].

Sustainable implementation ultimately depends on multidisciplinary governance [39]. AI systems must be developed and deployed through collaboration among clinicians, nurses, engineers, ethicists, and administrators to ensure technical robustness as well as ethical and operational acceptability [30]. Without such integration, even high-performing algorithms may remain confined to experimental prototypes rather than becoming trusted clinical tools [50].

Global best practices in AI-driven critical care

Global experience suggests that successful AI implementation in ICUs depends less on algorithmic sophistication and more on the surrounding clinical and organisational ecosystem [33]. Effective adoption requires alignment between technological capability, clinician engagement, and institutional readiness [15]. Context-sensitive deployment, supported by collaboration among clinicians, nurses, data scientists, and administrators, has been associated with improvements in workflow performance and patient outcomes [21].

Co-development with end users enhances clinical relevance, usability, and credibility of AI systems [9]. Participatory design ensures that AI tools are integrated into clinical reasoning rather than perceived as external impositions [26]. Local validation is equally critical, as models trained in one institutional or demographic context may not generalise reliably to another [35]. Periodic retraining with locally derived data can maintain performance, reduce bias, and strengthen clinician confidence [28]. Robust governance structures are essential for sustainability [40]. Ongoing performance monitoring, ethical auditing, and shared accountability mechanisms support transparency and early identification of bias or performance drift [17]. Open communication with healthcare professionals and patients regarding the role and limitations of AI further promotes responsible adoption and trust [30].

Successful integration also depends on compatibility with existing ICU workflows [43]. Systems that complement established practices without introducing disruptive structural changes are more likely to achieve sustained utilisation [8]. Overall, durable implementation requires coherence among people, processes, and policy rather than reliance on computational power alone [19]. Continuous ethical reflection, structured training, and iterative evaluation ensure that AI remains a clinically supportive and professionally accountable tool within critical care practice [41]. Table 3 summarises key elements associated with effective and sustainable AI integration in intensive care.

**Table 3:** Global Best Practices and Determinants of Successful AI Implementation in Intensive Care Units ICU: intensive care unit.

Best Practice	Implementation Approach	Observed Impact	Key Enablers / Requirements	Reference(s)
Local Contextual Adaptation	Designing AI solutions aligned with institutional and regional healthcare needs	Improves model relevance, clinical accuracy, and usability	Local data integration; stakeholder input	[15]
End-User Co-Development	Collaboration between developers, clinicians, and nurses during design and testing	Enhances usability, trust, and workflow alignment	Participatory design; iterative feedback	[9]
Validation with Locally Derived Data	Continuous model retraining using site-specific datasets	Maintains accuracy, minimises bias, and ensures equity	Access to representative data; regular updates	[28]
Ethical Governance and Oversight	Establishing multidisciplinary audit and review frameworks	Promotes transparency, accountability, and ethical compliance	Cross-functional committees; algorithmic audits	[17]
Transparent Communication and Trust Building	Engaging clinicians and patients in understanding AI outputs	Encourages responsible adoption and shared decision-making	Explainable interfaces; user education	[30]
Workflow Integration and Sustainability	Embedding AI tools into existing ICU systems and clinical routines	Improves efficiency, continuity, and adoption stability	Interoperable EHR platforms; staff training	[8]
Continuous Learning and Ethical Reflection	Incorporating education, evaluation, and ethical awareness into implementation cycles	Ensures long-term sustainability and compassionate use	Lifelong learning; reflective governance culture	[41]

Limitations and future recommendations

Although AI is portrayed as having great potential to improve the decision-making process in intensive care units, the existing evidence is limited by both methodological and practical constraints. Current literature shows a great diversity in the algorithms, datasets, and validation criteria that impedes significant comparison and reproducibility. Most studies are based on retrospective or single-centre data, which restricts the extent to which the study can be generalised to other populations, whereas the number of prospective and randomised studies remains small and cannot provide conclusive evidence on clinical effectiveness. Also, the publication bias in favour of positive outcomes and the high speed of obsolete algorithms in the face of technological change would pose a problem to longitudinal assessment. All these contribute to the importance of the standardisation of research designs and uniform reporting frameworks to create trustworthy standards of AI performance in critical care.

Moving forward, the challenge of future studies should be focused on elaborating explainable AI frameworks, which are ethically sound and which comply with clinical and patient safety standards. By including AI literacy in medical and nursing courses, the clinicians will be able to analyse the results of algorithms critically and responsibly. The emerging regulatory frameworks should define the responsibility between human decision-makers and intelligent systems to make the governance transparent. Innovation into equitable practice will require multidisciplinary cooperation among the clinicians, engineers, and ethicists. Continued assessment and refinements will eventually define how AI can be developed into a collaborative tool or an ally in enhancing critical-care processes.

## Conclusions

AI is transforming the nature of critical care by changing the way clinicians and nurses perceive complex data, forecast patient outcomes, and make high-stakes decisions. This revision offers a very useful synthesis, which is a unique mix of technology, ethical, and professional views, which are normally considered separately in the fields of nursing as well as in medicine. It makes a point that artificial intelligence is not about work automation, but rather its actual potential suggests the increase in human expertise, improved diagnostic accuracy, predictive precision, and coordination of workflows without loss of compassion and responsibility. This review offers a comprehensive picture of how human-AI interaction in an intensive care unit can be viewed by reviewing AI implications on a clinical, ethical, and interprofessional level. The results point to the fact that effective implementation requires elucidable, clear-cut, and context-specific systems that are backed by ethical governance and interdisciplinary participation. A safe adoption will involve equipping professionals in the field of healthcare with AI literacy and critical appraisal skills. In the end, this review contributes to the discussion by conceptualising AI as a tool and as a transformative companion, which, in combination with human empathy and ethical care, can help to redefine excellence in patient-centred critical care.
